# Synergistic Stabilization of Potassium Metal Anodes Through Orange‐Peel Elimination and Robust Solid–Electrolyte Interphase Formation

**DOI:** 10.1002/advs.75878

**Published:** 2026-05-29

**Authors:** Pan He, Yupei Han, Yundong Zhou, Ruhan He, Rui Xu, Yunlong Zhao, Junjun Wang, Wanjun Ren, Matthew Hopper, Qinyou An, Denys Makarov, Ian S. Gilmore, Liqiang Mai, Yang Xu

**Affiliations:** ^1^ State Key Laboratory of Advanced Electromagnetic Technology School of Electrical and Electronic Engineering Huazhong University of Science and Technology Wuhan Hubei China; ^2^ Department of Chemistry University College London London UK; ^3^ National Physical Laboratory Hampton UK; ^4^ Department of Engineering University of Cambridge Cambridge UK; ^5^ Helmholtz‐Zentrum Dresden‐Rossendorf e.V. Institute of Ion Beam Physics and Materials Research Dresden Germany; ^6^ Dyson School of Design Engineering Imperial College London London UK; ^7^ State Key Laboratory of Advanced Technology for Materials Synthesis and Processing Wuhan University of Technology Wuhan Hubei China

**Keywords:** metal anodes, orange‐peel effect, potassium batteries, solid‐electrolyte interphase, Surface roughness

## Abstract

Potassium (K) metal anodes are attractive for next‐generation rechargeable batteries due to their low redox potential and elemental abundance, yet their practical application is hindered by dendrite growth and unstable solid‐electrolyte interphases (SEIs). Here, we replace conventional roll pressing with a cutting‐based fabrication strategy to produce crystallographically more uniform and minimally deformed K surfaces, while independently tuning SEI chemistry through electrolyte concentration. This approach establishes a unified framework that elucidates the synergistic coupling between surface uniformity and SEI robustness in governing K plating/stripping stability. Only their synergy delivers fast kinetics, high areal capacity, and long‐term reversibility. Consequently, optimized K||K symmetric cells operate stably for over 4 000 h at 0.5 mA cm^−2^ and 4 mA h cm^−2^. Full cells paired with K_1.97_Mn[Fe(CN)_6_] cathodes retain 90% capacity after 1200 cycles. These findings highlight the importance of concurrent morphological and interfacial regulation for practical K metal anodes.

## Graphical abstract

Synergistic control of surface uniformity and solid–electrolyte interphase robustness regulates potassium deposition, suppressing dendritic growth and enabling uniform, dense potassium plating. This combined strategy promotes stable interfacial chemistry and homogeneous electric‐field distribution, leading to enhanced reversibility and long‐term cycling stability of potassium metal anodes.

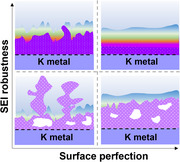

## 1 Introduction

Metal batteries (MBs) offer a compelling pathway toward next‐generation energy storage because of the exceptionally high energy densities enabled by metal anodes [1, 2, 3]. However, their practical deployment is severely limited by dendritic plating and stripping, which originate from non‐uniform electric fields and heterogeneous ion flux at the metal–electrolyte interface [4, 5]. These instabilities are closely associated with surface imperfections, including high roughness, microscopic protrusions, and fragile or chemically unstable SEIs [6, 7, 8, 9]. Once initiated, dendrite growth accelerates SEI rupture, electrolyte consumption, dead‐metal formation, and eventual short circuiting [10, 11].

Substantial efforts have been devoted to stabilizing metal anodes through surface engineering or SEI design [12, 13, 14, 15, 16, 17, 18]. Although each strategy can improve performance, their effectiveness is often undermined by poor reproducibility [19, 20, 21, 22]. This limitation arises because surface morphology and SEI chemistry are commonly treated as independent variables, despite being intrinsically coupled [23, 24, 25]. As a result, optimizing one factor without controlling the other frequently leads to unstable or irreproducible behavior.

For alkali metals, surface morphology is particularly sensitive to fabrication history [7, 26]. Roll pressing is widely used to prepare Na and K metal foils due to its simplicity [27, 28, 29], yet the mechanistic impact of roll‐induced plastic deformation on surface structure and electrochemical stability remains insufficiently understood. Potassium metal batteries (PMBs), which combine high energy density with favorable elemental abundance [30, 31, 32, 33], therefore provide an ideal platform to examine how manufacturing processes influence surface architecture and how surface morphology and SEI chemistry act together to govern plating and stripping behavior.

Here, we show that conventional roll pressing inevitably introduces severe plastic deformation into K metal, producing an “orange‐peel” surface that amplifies electric‐field heterogeneity and accelerates failure. To overcome this limitation, we introduce a cutting‐based fabrication strategy that yields smooth, crystallographically more uniform K surfaces with reduced deformation. By independently tuning SEI chemistry through electrolyte concentration, we demonstrate a synergistic coupling between surface uniformity and SEI robustness that enables dense, uniform K deposition and long‐term stability. This integrated approach extends K metal cycling beyond 4,000 h and establishes manufacturing‐aware interfacial design as a critical principle for stable metal batteries.

## 2 Results and Discussion

### 2.1 Comparison between Rolling and Cutting Manufacturing

**FIGURE 1:**
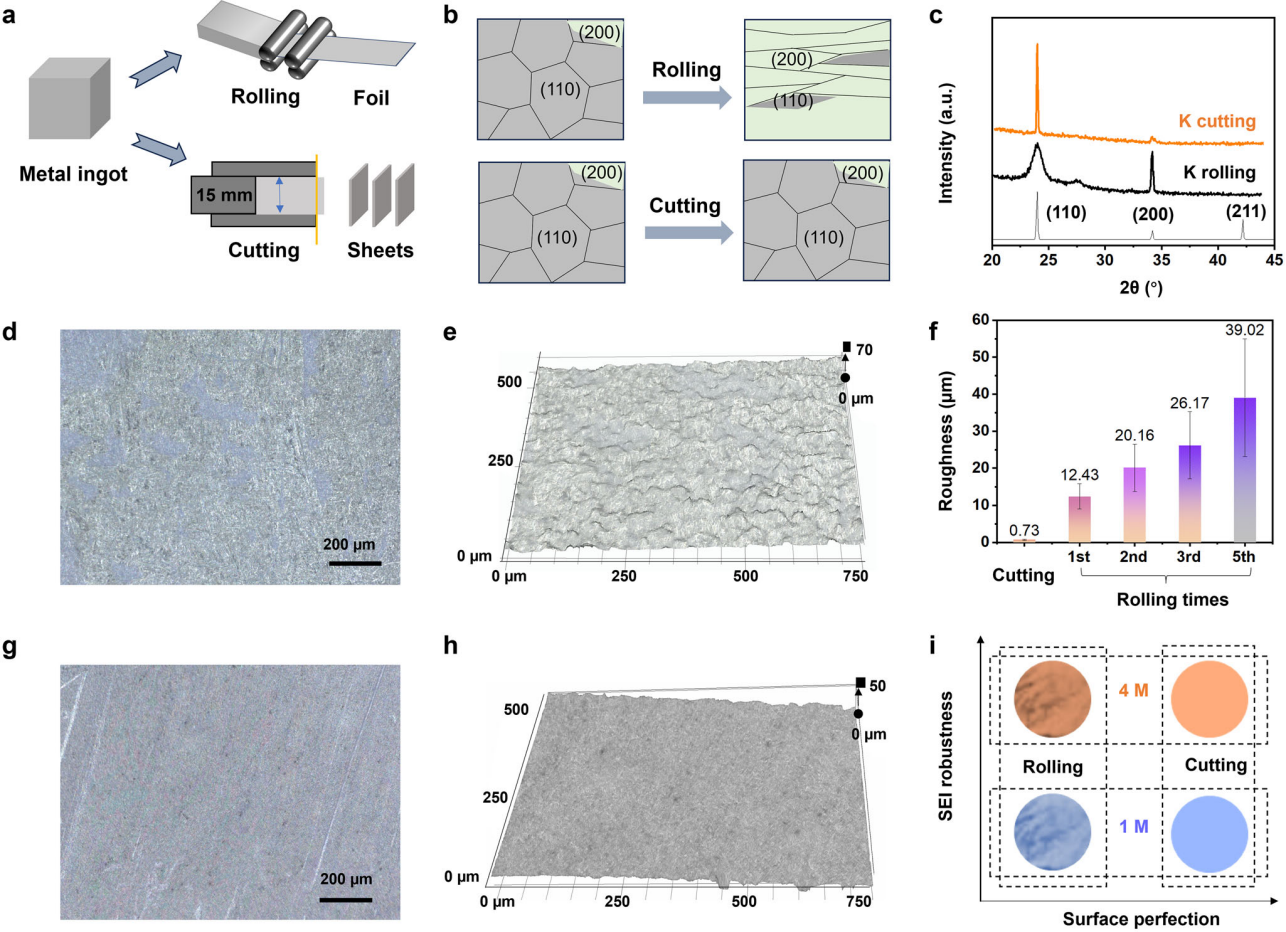
Comparative analysis of rolling and cutting processes for K metal anode fabrication. (a) Schematic illustration of deformation‐driven rolling and subtractive cutting processes used to prepare K metal electrodes. (b) Crystallographic schematics illustrating that rolling induces lattice distortion, grain elongation, and strain‐driven lattice rotation, whereas cutting largely preserves grain integrity and crystallographic homogeneity. (c) XRD patterns of K@rolling and K@cutting, where broadening and weakening of the (110) reflection, together with enhancement of the (200) reflection, indicate defect accumulation and strain‐induced texture evolution. (d,e) Optical and three‐dimensional confocal images of K@rolling reveal pronounced surface undulations and a characteristic “orange‐peel” morphology resulting from deformation‐induced grain mismatch. (f) Quantitative comparison of surface roughness, showing progressive roughening with repeated rolling and an approximately seventeen‐fold reduction in Ra for K@cutting. (g,h) Optical and three‐dimensional confocal images of K@cutting, demonstrating a flat and uniform surface without rolling marks or microscale deformation features. (i) Schematic summary of the four representative samples (K@rolling‐1 M, K@rolling‐4 M, K@cutting‐1 M, and K@cutting‐4 M) used to decouple the effects of surface morphology and SEI robustness on K plating and stripping behavior.

To elucidate how manufacturing routes dictate the surface properties of K metal anodes, we systematically compared deformation‐driven rolling with a subtractive cutting process (Figure 1a). In conventional rolling, a K ingot is repeatedly flattened between steel rollers to form thin foils and punched into discs (denoted as K@rolling). This process inevitably subjects the soft K metal to severe plastic strain. As deformation accumulates, the crystal lattice undergoes non‐uniform distortion, leading to grain elongation and slip‐band formation (Figure 1b). These intragranular processes progressively increase mismatch at grain boundaries and ultimately generate surface ridges characteristic of deformation‐induced roughening. During rolling, strain is accommodated primarily through shear on low‐index BCC slip planes and associated directions, which drives lattice rotation and reorients a fraction of grains so that their {100} planes become preferentially aligned perpendicular to the foil surface. This crystallographically guided plastic flow amplifies surface undulations and gives rise to the macroscopic “orange‐peel” texture.

These deformation‐induced features are corroborated by XRD (Figure 1c). The rolled sample exhibits a broadened and weakened (110) reflection, consistent with defect accumulation and microstrain on the primary slip planes [34], accompanied by a pronounced enhancement of the (200) peak, which is indicative of possible strain‐induced texture evolution. A weak feature near ∼27.5° is also observed, which can be tentatively assigned to K_2_O [35], suggesting slight surface oxidation. Optical and 3D confocal microscopy (Figure 1d,e) further reveal a highly corrugated surface, typical of strain localization and intergranular relief in cold‐worked metals [36]. As a result, the rolled K surface displays a high roughness (Ra ≈ 39 µm), which correlates with increased interfacial resistance and reduced electrochemical stability (Figure S1).

In contrast, the cutting process is purely subtractive: the K ingot is sliced with a sharp blade to remove surface layers without inducing significant bulk deformation (Figure S2). The resulting K@cutting surface is flat and uniform, free of rolling marks and microscale undulations (Figure 1g,h). Because mechanical deformation is minimized, the underlying grains remain largely intact and retain a high degree of crystallographic uniformity. Consequently, the surface roughness is reduced by approximately 17‐fold compared with rolled K (Figure 1f).

With this clear morphological contrast established, electrolytes with different KFSI concentrations (1 M and 4 M) were employed to generate SEIs of comparable chemistry but distinct robustness (Figure S3) [37, 38], enabling systematic evaluation of the coupled roles of surface morphology and SEI stability [39, 40]. Four representative electrodes, K@rolling‐1 M, K@rolling‐4 M, K@cutting‐1 M, and K@cutting‐4 M, were thus prepared for subsequent plating and stripping studies (Figure 1i).

### 2.2 Electrochemical Behaviors of K Plating/Stripping

**FIGURE 2:**
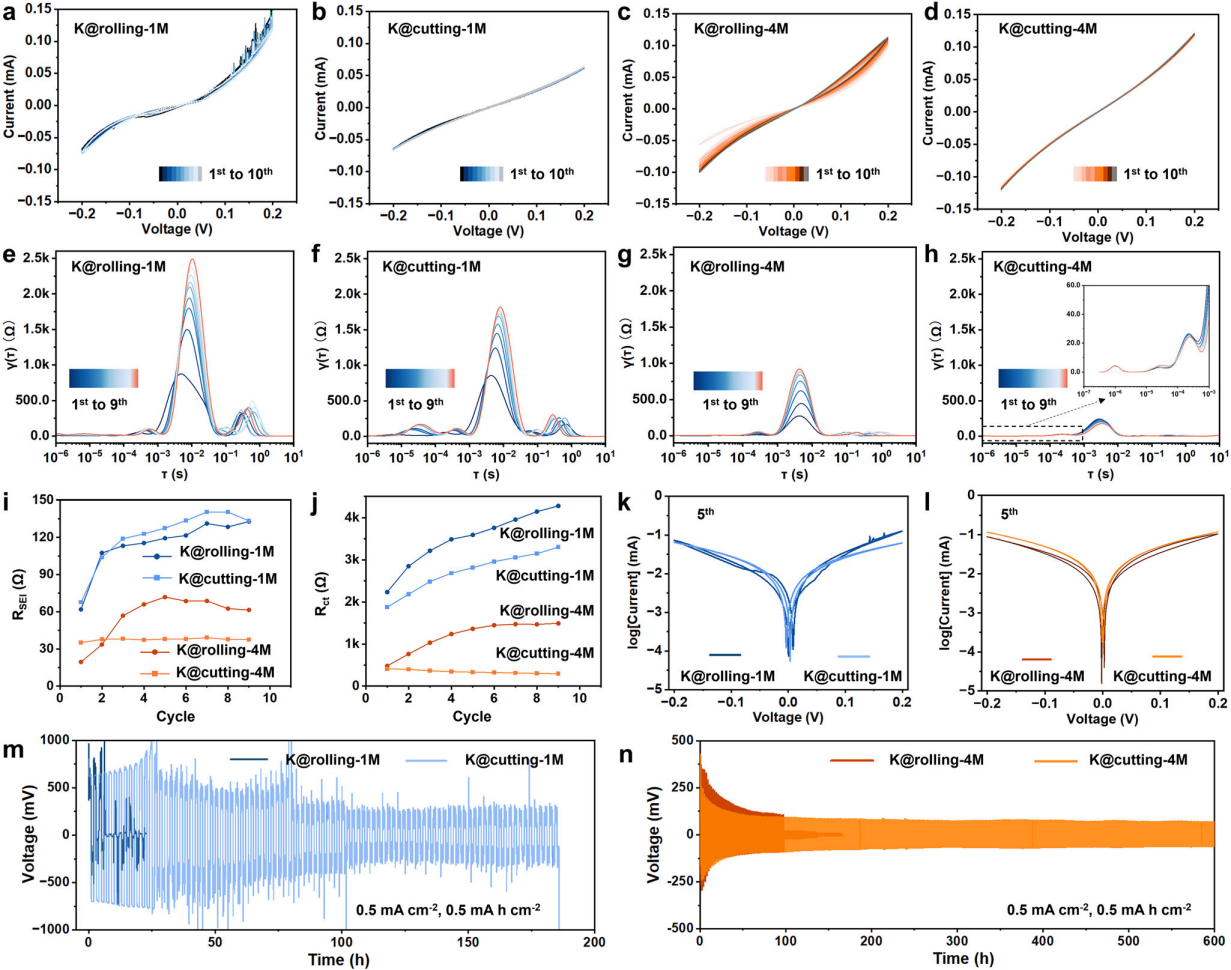
Electrochemical characterization of K plating and stripping behavior. Cyclic voltammetry (CV) profiles recorded over 10 cycles for (a) K@rolling‐1 M, (b) K@cutting‐1 M, (c) K@rolling‐4 M, and (d) K@cutting‐4 M symmetric cells. (e–h) Distribution‐of‐relaxation‐time (DRT) spectra derived from electrochemical impedance spectroscopy, resolving contributions associated with SEI ion transport and interfacial charge transfer for the corresponding electrodes. (i,j) Cycle‐dependent evolution of SEI resistance (R_SEI_) and charge‐transfer resistance (R_ct_) extracted from DRT analysis over nine cycles. (k,l) Tafel plots obtained at the fifth cycle, highlighting differences in interfacial kinetics among the four electrodes. (m,n) Long‐term galvanostatic cycling performance of symmetric cells at 0.5 mA cm^−^^2^ and 0.5 mA h cm^−^^2^, showing the lowest polarization and superior cycling stability for K@cutting‐4 M.

The electrochemical responses of the four electrodes (K@rolling‐1 M, K@cutting‐1 M, K@rolling‐4 M, and K@cutting‐4 M) are summarized in Figure 2, with complementary impedance analysis provided in Figure S4. Cyclic voltammetry (CV) was performed in symmetric K||K cells at 1 mV s^−^^1^ within a voltage window of −0.2 to 0.2 V for ten consecutive cycles. As shown in Figure 2a–d, K@rolling‐1 M exhibits pronounced current fluctuations and distorted CV profiles, indicative of unstable and poorly reversible K plating and stripping. In contrast, K@cutting‐1 M displays smooth and nearly overlapping anodic and cathodic curves, reflecting improved reversibility enabled by its more uniform surface. Increasing the electrolyte concentration to 4 M enhances the overall current response for both rolled and cut electrodes, consistent with accelerated K^+^ transport. Notably, only K@cutting‐4 M maintains fully symmetric and stable CV profiles throughout cycling, underscoring that reversible K redox behavior requires the simultaneous presence of surface uniformity and a robust SEI.

Electrochemical impedance spectroscopy combined with distribution‐of‐relaxation‐time (DRT) analysis (Figure 2e–j, extended in Figure S4) provides further insight into the coupled roles of surface morphology and SEI chemistry. Two characteristic relaxation processes are resolved: a high‐frequency component associated with ionic transport through the SEI (R_SEI_) and a low‐frequency component corresponding to charge transfer at the K/SEI interface (R_ct_) [41, 42]. In the 1 M electrolyte, both K@rolling‐1 M and K@cutting‐1 M exhibit pronounced DRT peaks that intensify and shift toward longer relaxation times with cycling (Figure 2e,f), indicating progressive SEI thickening and increasingly sluggish charge transfer [43, 44, 45]. Quantitatively, R_SEI_ and R_ct_ in K@rolling‐1 M increase from ∼57 and ∼2233 Ω to ∼133 and ∼4281 Ω by the nineth cycle. Although K@cutting‐1 M shows slightly lower values (around 131 and 3301 Ω), significant impedance growth is still observed, demonstrating that surface smoothing alone cannot prevent interfacial degradation when the SEI is intrinsically unstable.

In contrast, the impedance evolution is markedly suppressed in the 4 M electrolyte. K@rolling‐4 M exhibits only moderate growth in R_ct_, increasing from approximately 479 to 1490 Ω, whereas K@cutting‐4 M maintains nearly constant and minimal resistances over nine cycles, with R_SEI_ ≈ 38 Ω and R_ct ≈ 350 Ω (Figure 2g,h and enlarged view in Figure S5). The evolution curves in Figure 2i,j clearly illustrate this divergence: both R_SEI_ and R_ct_ rise steadily for the 1 M systems, while they remain essentially unchanged for K@cutting‐4 M. This behavior reflects the formation of a compact, inorganic‐rich SEI together with a crystallographically homogeneous and defect‐minimized K surface that supports stable ion transport and charge transfer.

Kinetic parameters extracted from Tafel plots (Figure 2k,l and Figure S6 and Table S1) are consistent with these trends. The exchange current density j_0_ increases from approximately 2.5 × 10^−^^3^ mA cm^−^^2^ for K@rolling‐1 M to ∼4.1 × 10^−^^3^ mA cm^−^^2^ for K@cutting‐4 M, indicating progressively accelerated interfacial kinetics. In line with these kinetic improvements, long‐term symmetric‐cell cycling (Figure 2m,n) shows that electrodes with either rough surfaces or weak SEIs fail rapidly. In the 1 M electrolyte, K@rolling‐1 M short‐circuits within hours, while K@cutting‐1 M fails after about 180 h due to continuous SEI degradation. Increasing electrolyte concentration improves the stability of K@rolling‐4 M, but surface‐roughness‐induced deposition inhomogeneity still limits cycle life. By contrast, K@cutting‐4 M combines a deformation‐minimized surface with a chemically robust SEI and sustains low polarization (approximately 250 mV) with stable cycling for more than 600 h. Collectively, these results demonstrate that surface uniformity and SEI robustness must act cooperatively to suppress dendrite formation and enable long‐term reversible K metal cycling.

### 2.3 Surface Morphology, Electric‐Field Distribution, and Interfacial Evolution

**FIGURE 3:**
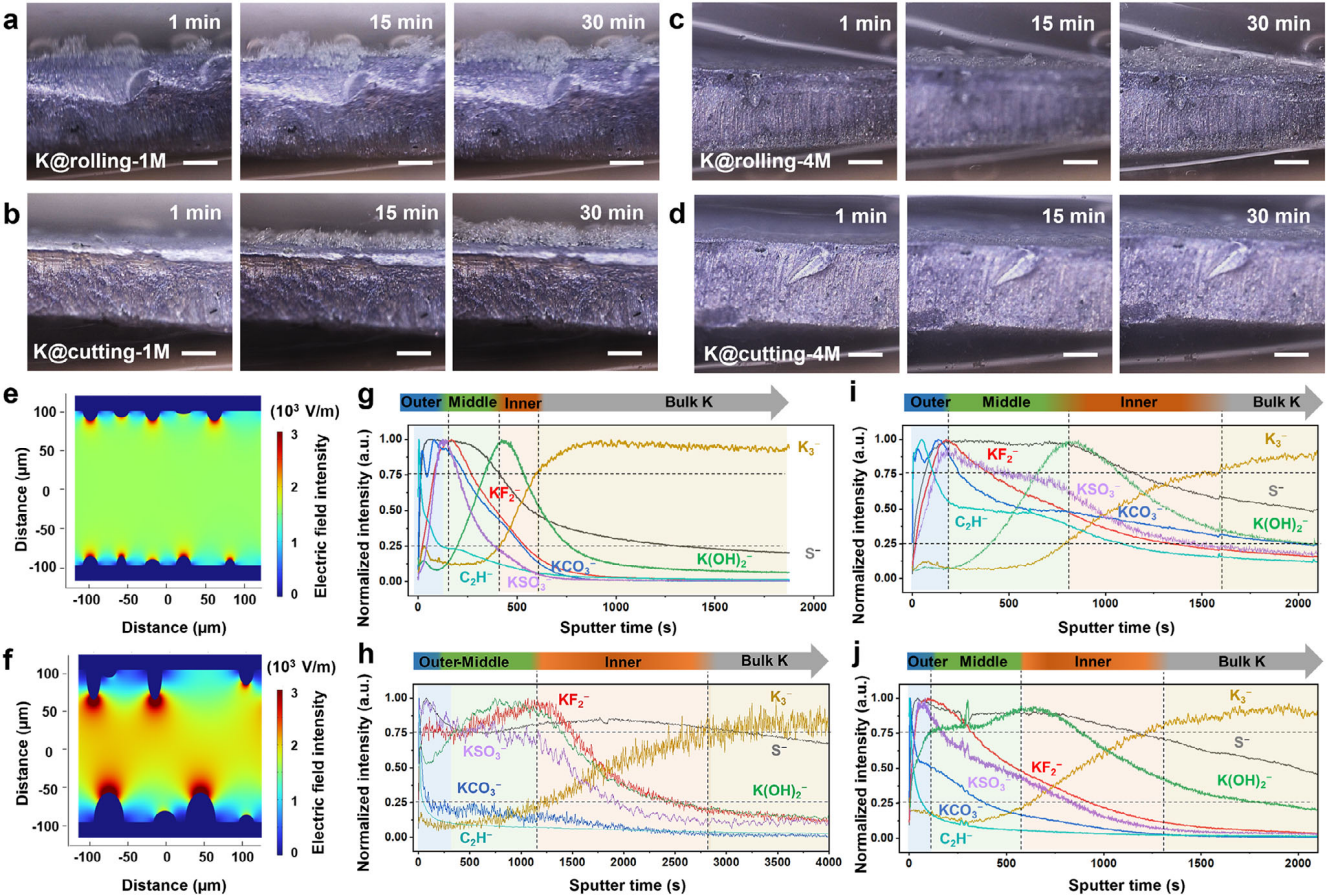
Correlation between surface morphology, electric‐field distribution, and interfacial evolution of K electrodes. (a–d) In situ optical microscopy images of K@rolling and K@cutting electrodes in 1 and 4 M electrolytes recorded at 1, 15, and 30 min during plating (scale bars: 200 µm); extended observations at 60 min are shown in Figure S7. (e,f) COMSOL‐simulated electric‐field distributions comparing field localization on rough K surfaces with uniform field distributions on smooth K surfaces. (g–j) ToF‐SIMS depth profiles of SEIs on K@cutting‐1 M and K@cutting‐4 M before and after 10 cycles, highlighting the transition from a thin, solvent‐derived SEI (1 M) to a thick, salt‐derived, mechanically stable SEI (4 M) with preserved multilayer structure.

To elucidate how electrode morphology and SEI robustness cooperatively regulate the spatiotemporal dynamics of K deposition, in situ optical microscopy was performed over extended plating intervals (1–60 min) at 0.5 mA cm^−^^2^ using transparent home‐made symmetric cells (Figure 3a–d and Figure S7). In the 1 M electrolyte (Figure 3a), K@rolling‐1 M exhibits highly heterogeneous nucleation within the first minute, with protrusions emerging at deformation‐induced asperities. These perturbations amplify rapidly. By 15 min, dendritic structures become pronounced, and thickness growth becomes strongly localized. By 30 min, the deposit evolves into a porous, filamentous network, characteristic of persistent interfacial instability governed by both surface roughness and fragile solvent‐derived SEI. In contrast, K@cutting‐1 M (Figure 3b) shows spatially uniform nucleation at 1 min, and although the deposit becomes loose at 15–30 min, its vertical growth remains homogeneous. The absence of protrusion sharpening demonstrates that eliminating deformation‐induced roughness suppresses field amplification and delays the onset of morphological instability.

A distinctly different regime arises in the 4 M electrolyte (Figure 3c,d). Both K@rolling‐4 M and K@cutting‐4 M exhibit compact and laterally continuous nucleation fronts, reflecting the stabilizing influence of a salt‐derived, inorganic‐rich SEI. At 15 min, K@rolling‐4 M develops mild undulations while remaining structurally coherent; K@cutting‐4 M maintains the densest and most uniform deposits, with negligible perturbation even at 30 min. Extending plating to 60 min (Figure S6) further reinforces this trend: the synergy between a smooth surface and a robust SEI yields the most compact and morphologically ordered K deposits, while rough surfaces or weak SEIs invariably promote dendritic evolution.

COMSOL simulations of the surface electric field (Figure 3e,f) directly support these findings. The rolled K surface concentrates electric fields at surface ridges, creating localized hotspots that drive dendritic initiation. Conversely, cutting produces a smooth, crystallographically homogeneous surface with spatially uniform fields and ion flux, thereby suppressing perturbation growth. Together, these results establish that K deposition stability is jointly governed by surface‐derived electric‐field uniformity and electrolyte‐determined SEI integrity, and that their synergy is essential for suppressing long‐wavelength and short‐wavelength morphological instabilities over extended plating durations.

Complementary ToF‐SIMS depth profiling further clarifies how electrolyte concentration dictates SEI composition and architecture (Figure 3g–j and Figure S8). To minimize topographic artefacts [46], K@cutting‐1 M and K@cutting‐4 M were selected. In both electrolytes, solvent‐derived organics (detected by C_2_H^−^) and salt‐derived inorganics (KF_2_^−^, KSO_3_^−^, S^−^, KCO_3_^−^, and K(OH)_2_^−^) coexist [38, 47], accompanied by K_2_CO_3_ and KOH formed from correlated decomposition pathways [38, 48, 49]. Depth‐resolved profiles reveal a consistent three‐layered SEI structure consisting of an organic‐rich outer layer, an inorganic‐rich middle layer, and an inorganic‐dominated inner layer adjacent to bulk K (marked by K_3_^−^). However, the thickness, integrity, and chemical gradients of these layers depend strongly on electrolyte concentration.

Before cycling (Figure 3g,i), K@cutting‐1 M displays a thin outer organic layer and limited inorganic accumulation, whereas K@cutting‐4 M forms substantially thicker middle and inner layers enriched in KF, K_2_SO_3_, K_2_S, and KOH, indicating stronger salt decomposition and a denser inorganic matrix. After 10 cycles, K@cutting‐1 M undergoes marked structural degradation: the outer thins, the middle layers and inner layer thicken due to inorganic buildup, and the K_3_^−^ signal becomes attenuated, consistent with repeated SEI rupture and reconstruction (Figure 3h). In sharp contrast, K@cutting‐4 M preserves its layered architecture, maintaining distinct boundaries and uniformly distributed inorganic species even after cycling (Figure 3j). 2D ion‐distribution maps (Figure S8) corroborate this conclusion: K@cutting‐1 M displays weakened and disordered signals after cycling, whereas K@cutting‐4 M maintains uniform spatial distributions. Together, these morphological and chemical analyses demonstrate that a high‐concentration electrolyte yields a thick, inorganic‐rich, mechanically resilient SEI that resists cycling‐induced disruption, supports uniform K^+^ transport, and enables smooth, dendrite‐free deposition. When combined with a deformation‐free K surface, the resulting synergy produces the most stable plating morphology and the most robust interfacial chemistry.

**FIGURE 4:**
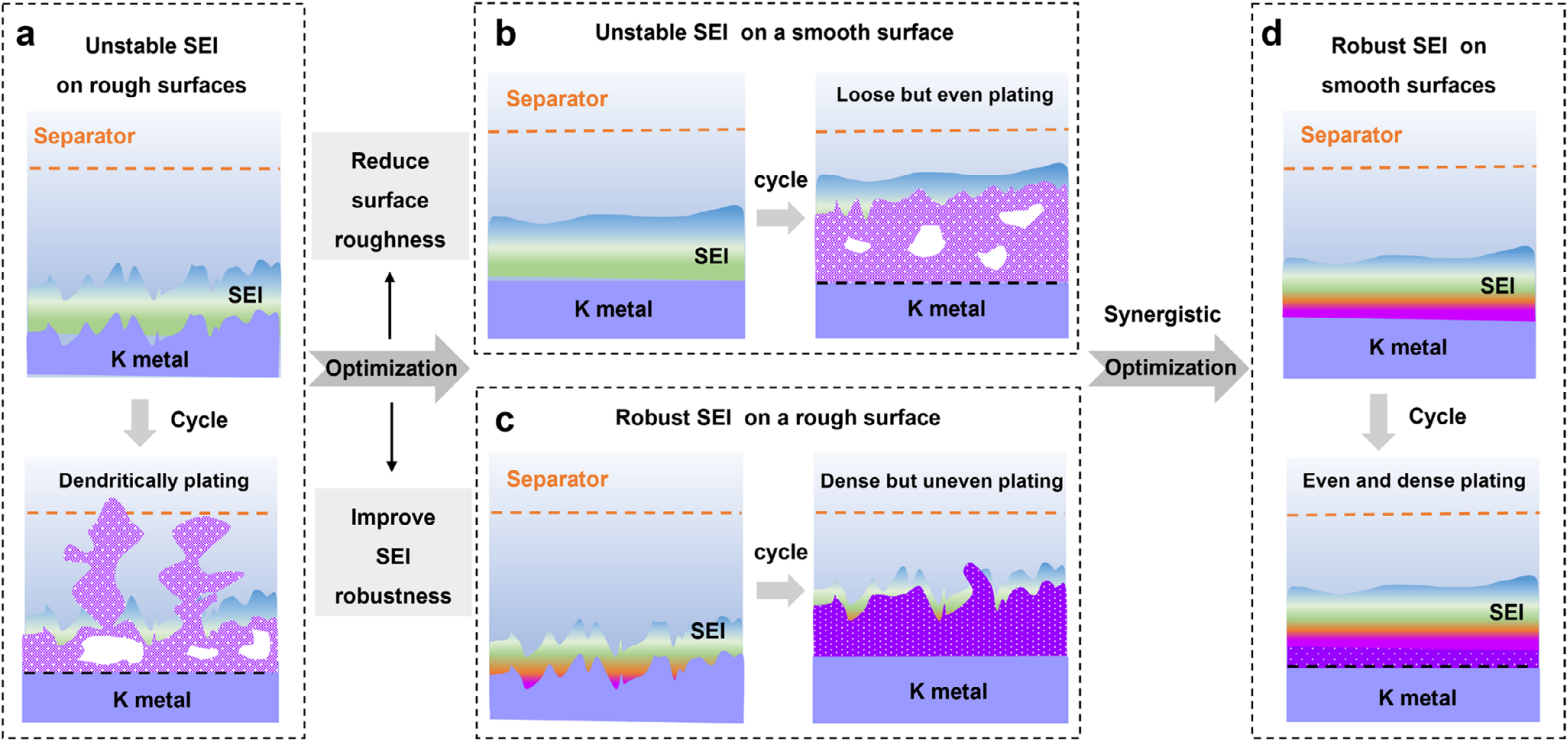
Schematic illustration of synergistic optimization of K stripping/plating by surface morphology and SEI robustness. (a) K@rolling‐1 M, combining a rough surface with a weak SEI, exhibits highly inhomogeneous and porous K deposition, leading to pronounced dendritic growth during cycling. (b) K@cutting‐1 M shows more spatially uniform deposition due to the smoother surface, but the deposits remain loosely packed because of insufficient SEI stability. (c) K@rolling‐4 M forms a chemically denser SEI; however, surface roughness still induces nonuniform deposition and localized dendrite growth. (d) K@cutting‐4 M integrates a smooth surface with a robust SEI, enabling dense, uniform K deposition and stable stripping/plating behavior.

To conclude, the comparative analysis makes clear that surface smoothness and SEI stability are the two primary determinants of cycling behavior in potassium metal anodes, and that their synergistic interaction amplifies the benefits of each individual factor. As summarized in Figure 4, the cutting process provides a much smoother and more homogeneous K surface than rolling, while the high‐concentration electrolyte forms a mechanically robust SEI that is intrinsically more resistant to dissolution and fracture. When both parameters are non‐ideal conditions, as in the K@rolling‐1 M sample (Figure 4a), the combination of severe surface roughness and a fragile, solvent‐dominated SEI accelerate electrolyte decomposition, promotes accumulation of inorganic fragments, initiates dendritic protrusions, and leads to cell failure within only a few cycles. Improving the surface alone, as in K@cutting‐1 M (Figure 4b), mitigates these instabilities but cannot eliminate them; the weak SEI still thickens and fractures during cycling, causing rising impedance, uneven deposition, and eventual short‐circuit failure after ∼180 h. Enhancing only the SEI, as in K@rolling‐4 M (Figure 4c), improves chemical stability, but roughness‐induced field amplification persists, leading to nonuniform plating and limiting lifespan to ∼100 h. In contrast, the K@cutting‐4 M electrode (Figure 4d), which benefits from both a smooth surface and a robust, inorganic‐rich SEI, preserves structural integrity, supports dense and uniform deposition, and enables markedly extended cycling stability. These results demonstrate that the concurrent regulation of surface morphology and SEI chemistry is indispensable for realizing durable, dendrite‐free K metal anodes and position K@cutting‐4 M as a particularly promising strategy for practical applications.

### 2.4 Enhanced Performance of K@cutting‐4 M

**FIGURE 5:**
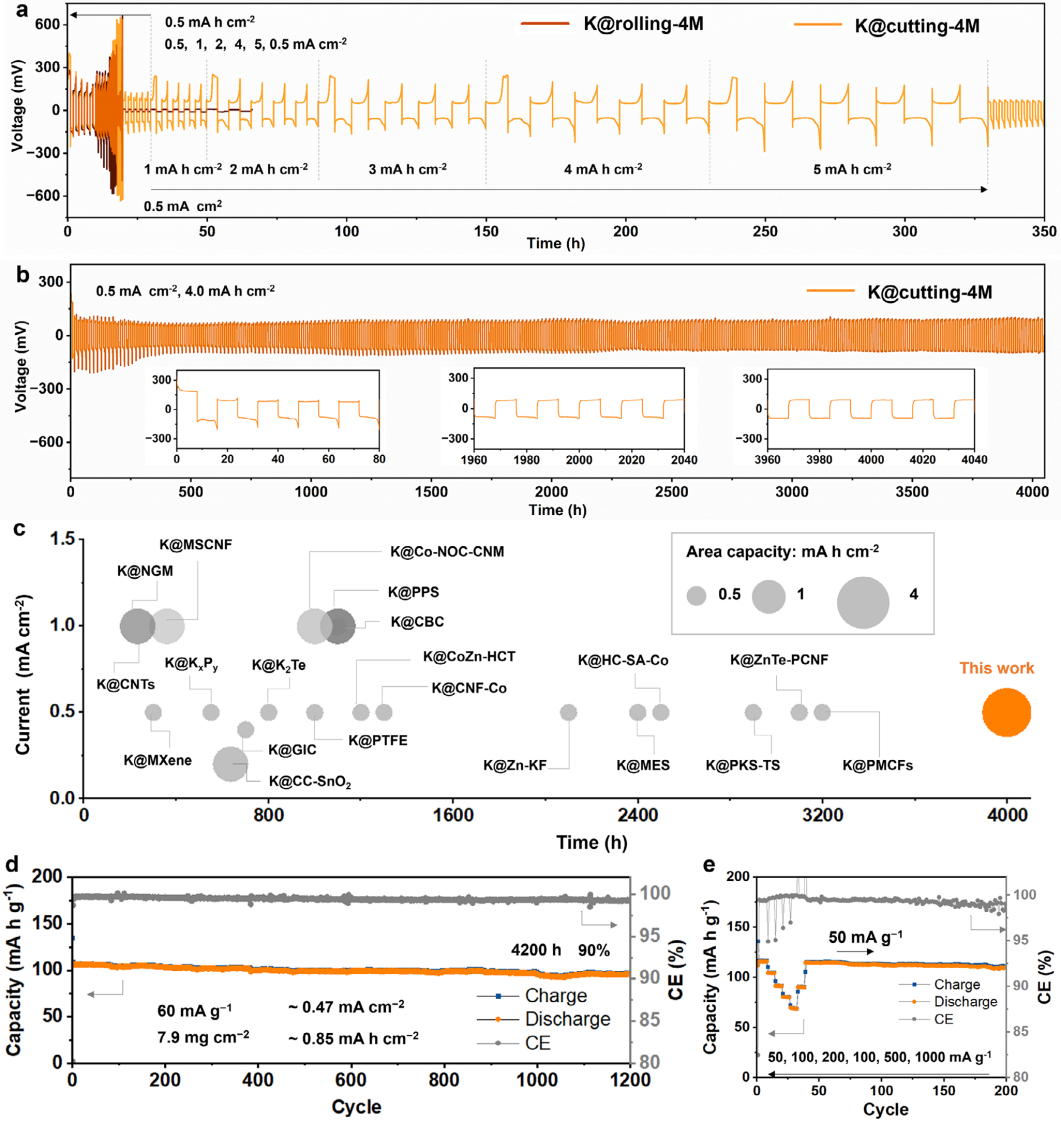
Electrochemical performance of potassium metal batteries. (a) Galvanostatic cycling profiles of symmetric K batteries: rate performance at current densities of 0.5, 1, 2, 3, and 5 mA cm^−2^ with a fixed areal capacity of 0.5 mA h cm^−2^, and at 0.5 mA cm^−2^ with varying areal capacities of 1, 2, 3, 4, and 5 mA h cm^−2^. (b) Long‐term cycling stability at 0.5 mA cm^−2^ with a capacity of 4 mA h cm^−2^. The inset highlights the voltage profiles at the beginning, middle, and end of cycles over 80 h of cycling. (c) The Comparison of the K||K cycle life between the K@cutting‐4 M and the reported K metal anodes. (d) Long‐term cycling stability of the K||K_1.97_Mn[Fe(CN)_6_] full cell at a high current density of 0.47 mA cm^−2^ and high capacity of 0.85 mA h cm^−2^. (e) Rate capability of the K||K_1.97_Mn[Fe(CN)_6_] full cell measured at current densities ranging from 50 to 1000 mA g^−1^.

To further validate the enhanced electrochemical performance of K@cutting‐4 M, symmetric K||K cells were evaluated under stringent cycling conditions involving both high current densities and large plating capacities. As shown in Figure 5a, when the current density was increased stepwise from 0.5 to 5 mA cm^−^^2^ at a fixed areal capacity of 0.5 mA h cm^−^^2^, K@rolling‐4 M failed rapidly upon reaching 4 mA cm^−^^2^ and completed less than 20 h of cycling. In stark contrast, K@cutting‐4 M remained stable even at 5 mA cm^−^^2^, indicating its excellent tolerance to fast K^+^ transport. Similarly, when the areal capacity was increased from 0.5 to 5 mA h cm^−^^2^ at a constant 0.5 mA cm^−^^2^, K@cutting‐4 M maintained smooth voltage profiles without detectable short‐circuiting. This robustness highlights the cooperative effect of its uniform surface and mechanically stable SEI in enabling homogeneous K plating/stripping. Notably, during long‐term cycling at 0.5 mA cm^−^^2^ with a large plating capacity of 4 mA h cm^−^^2^, K@cutting‐4 M sustained more than 4,000 h of continuous operation with a low stable hysteresis of <300 mV (Figure 5b). This performance surpasses most previously reported K metal anodes (Figure 5c and Table S2), underscoring its interfacial resilience under realistic operational conditions.

Beyond symmetric cells, full‐cell testing employed K_1.97_Mn[Fe(CN)_6_] (see Materials and Methods, Figure S9 and Table S3) as the cathode with a high mass loading of 7.9 mg cm^−^^2^ (0.85 mA h cm^−^^2^). The charge/discharge profiles from the first to 1200th cycle at 100 mA g^−^^1^ (Figure S10) show excellent overlap, confirming high reversibility. As shown in Figure 5d, the K@cutting‐4 M full cell delivered an initial 107 mA h g^−^^1^, stabilized near 100 mA h g^−^^1^, and retained 90% capacity after 1,200 cycles with an average CE of 99.42%. By contrast, the K@rolling‐4 M full cell exhibited capacity fluctuations after 200 cycles and failed before 400 cycles (Figure S11). Furthermore, the rate‐capability analysis (Figure 5e) demonstrates reversible capacities of 112, 103, 90, 78, and 65 mA h g^−^^1^ at 50–1000 mA g^−^^1^, with full recovery to 112 mA h g^−^^1^ upon returning to 50 mA g^−^^1^, maintaining nearly 100% capacity retention over the subsequent 150 cycles.

## 3 Conclusions

In summary, this work elucidates the critical interplay between surface morphology and SEI robustness in stabilizing K metal anodes. The cutting‐based fabrication method produces a minimally deformed and more crystallographically uniform surface; concurrently, the 4 M KFSI/DEGDME electrolyte generates a dense, inorganic‐rich SEI. Their synergy dramatically enhances electrochemical stability, achieving performance that cannot be achieved by optimizing surface morphology or SEI chemistry in isolation. The optimized K@cutting‐4 M anode endures high currents (up to 5 mA cm^−^^2^), large capacities (up to 5 mA h cm^−^^2^), and exceptional long‐term cycling (>4000 h). Full cells pairing K@cutting‐4 M with a high‐loading Prussian‐blue‐analogue cathode deliver outstanding cycling stability and rate capability. Together, these findings highlight a practical and effective pathway toward stable, high‐energy‐density potassium metal batteries.

## Author Contributions

**Rui Xu**: methodology, validation, data curation, investigation, visualization, formal analysis, writing – review and editing. **Yundong Zhou**: methodology, data curation, formal analysis, visualization, writing – review and editing, investigation. **Yupei Han**: conceptualization, methodology, data curation, validation, investigation, formal analysis, writing – review and editing. **Liqiang Mai**: funding acquisition, writing – review and editing, supervision, resources. **Junjun Wang**: investigation, validation, writing – review and editing, formal analysis. **Wanjun Ren**: data curation, investigation, formal analysis, visualization, writing – review and editing. **Pan He**: conceptualization, methodology, data curation, investigation, validation, formal analysis, visualization, resources, writing – original draft, writing – review and editing. **Yunlong Zhao**: funding acquisition, writing – review and editing, resources, formal analysis. **Denys Makarov**: data curation, supervision, investigation, visualization, writing – review and editing, methodology, resources. **Qinyou An**: funding acquisition, writing – review and editing, investigation. **Matthew Hopper**: formal analysis, visualization, investigation, writing – review and editing. **Ian S. Gilmore**: methodology, data curation, validation, visualization, formal analysis, writing – review and editing, resources. **Ruhan He**: methodology, validation, writing – review and editing, formal analysis, investigation. **Yang Xu**: conceptualization, supervision, resources, project administration, formal analysis, validation, funding acquisition, writing – review and editing.

## Conflicts of Interest

The authors declare no conflicts of interest.

## Supporting information




**Supporting File**: advs75878‐sup‐0001‐SuppMat.docx.

## Data Availability

The data that support the findings of this study are available from the corresponding author upon reasonable request.
