# Supplementary material for: Synergistic Stabilization of Potassium Metal Anodes Through Orange‐Peel Elimination and Robust Solid–Electrolyte Interphase Formation
Source: Adv Sci (Weinh). 2026 May 29;13(47):e75878. doi: 10.1002/advs.75878 (PMC13336037; doi:10.1002/advs.75878)
Supplement: Supplementary file 1 [file ADVS-13-e75878-s001.docx]

Supporting Information

**Synergistic Stabilization of Potassium Metal Anodes through Orange-Peel Elimination and Robust Solid–Electrolyte Interphase Formation**

*Pan He,^1, 2^ Yupei Han,^2^ Yundong Zhou,^3^ Ruhan He,^4^ Rui Xu,^5^ Yunlong Zhao,^3,6^ Junjun Wang,^7^* *Wanjun Ren,^2^ Matthew Hopper,^2^ Qinyou An,^7^ Denys Makarov,^5^ Ian S. Gilmore,^3^ Liqiang Mai,^7, *^ Yang Xu^2, *^*

^1^ State Key Laboratory of Advanced Electromagnetic Technology, School of Electrical and Electronic Engineering, Huazhong University of Science and Technology, Wuhan, Hubei 430074, China

^2^ Department of Chemistry, University College London, London WC1H 0AJ, UK

^3^ National Physical Laboratory, Hampton, Teddington TW11 0LW, UK

^4^ Department of Engineering, University of Cambridge, Cambridge CB3 0FS, UK

^5^ Helmholtz-Zentrum Dresden-Rossendorf e.V., Institute of Ion Beam Physics and Materials Research, Bautzner Landstrasse 400, 01328, Dresden, Germany

^6^ Dyson School of Design Engineering, Imperial College London, London SW7 2DB, UK

^7^ State Key Laboratory of Advanced Technology for Materials Synthesis and Processing, Wuhan University of Technology, Hubei, Wuhan 430070, China

**Corresponding authors**: L.Q. Mai ([mlq518@whut.edu.cn](mailto:mlq518@whut.edu.cn)); Y. Xu (y.xu.1@ucl.ac.uk)

**Materials and Methods**

**Materials:** Potassium ingots (in mineral oil, 98% trace metals basis) were purchased from Fisher Scientific (Thermo, UK) and transferred to hexane (99%, anhydrous) for storage. Potassium trifluoromethanesulfonimide (KFSI, 99.5%) was purchased from Solvionic (France) and dried to remove water. Diethylene glycol dimethyl ether (DEGDME, 99.5%, anhydrous), Carbon black, poly(vinylidene fluoride) (PVDF), 1-methyl-2-pyrrolidone (NMP) were purchased from Sigma Aldrich. Glass firbre (GF/B) was purchased from Whatman.

**Preparation of the electrolyte:** The electrolyte was prepared by dissolving the KFSI salt in the DEGDME solvent at a concentration of 4 M.

**Preparation of K@rolling discs:** All operations were conducted in an Ar-filled glovebox (O_2_, H_2_O < 0.1 ppm) to prevent oxidation and moisture contamination. A bulk K chunk was first retrieved from mineral oil and gently wiped with dry Kimwipes to remove surface oil. The oxidized outer layer was then carefully scraped with a stainless-steel blade until a bright metallic luster was obtained, ensuring a clean metallic surface. The cleaned K block was subsequently roll-pressed between a stainless-steel roller and a flat steel plate to form a thin sheet. The pressing was performed in several gentle passes to gradually reduce the thickness to approximately 0.4–0.6 mm, while minimizing tearing or sticking of the soft metal. During the rolling process, the ductile K underwent plastic flow and surface stretching, leading to the formation of characteristic *“orange-peel”* textures with microscale undulations and uneven surface morphology. The rolled K plate was then punched into 12 mm-diameter discs using a precision punch die. The resulting K@rolling discs were immediately transferred into sealed PTFE containers and stored under Ar atmosphere before cell assembly.

**Preparation of K@cutting discs:** All procedures were carried out in an Ar-filled glovebox (O_2_, H_2_O < 0.1 ppm) to avoid contamination and oxidation. The cleaned K was then trimmed into a rectangular brick (~15 mm wide) on a PTFE cutting board. To ensure uniform slicing thickness, a home-made jig fabricated by 3D printing with PETG (polyethylene terephthalate glycol) polymer was employed. PETG was selected for its excellent mechanical strength, chemical resistance, and dimensional stability under glovebox conditions. The jig consisted of two parallel guide plates separated by a fixed gap (typically 0.4–0.6 mm) defining the target sheet thickness. The K brick was gently inserted into the jig and sliced along the guiding plane using a fresh stainless-steel blade to obtain smooth and uniform K sheets. Finally, 12 mm-diameter discs were punched from the sheets using a precision die and immediately stored in sealed PTFE containers under Ar.

This cutting-based approach represents a mechanically simple and potentially scalable subtractive process that can be readily integrated with controlled environments. Compared to conventional rolling, it minimizes plastic deformation and suppresses orange-peel surface roughness, while offering improved surface uniformity and reproducibility. Such a jig-assisted slicing strategy is also compatible with subsequent electrode processing steps, suggesting potential for practical implementation.

**Cathode**: K_1.97_Mn[Fe(CN)_6_] powders were synthesized using a conventional method.^1, 2^ Specifically, it was prepared via potassium citrate-assisted co-precipitation by dropwise adding a solution of 0.04 M MnCl_2_ and 0.2 M K_3_C_6_H_5_O_7_ (100 mL) into a solution of 0.04 M K_4_Fe(CN)_6_ and 0.2 M K_3_C_6_H_5_O_7_ (100 mL) over 2 h, followed by aging for 20 h. The resulting precipitate was collected by centrifugation, washed with deionized water and ethanol, and vacuum-dried at 60 °C. Microwave plasma atomic emission spectroscopy (MP-AES, Agilent 4210 MP-AES), and elemental analysis (Thermo Flash 2000) are carried out for chemical compositions. The slurry was prepared by mixing K_1.97_Mn[Fe(CN)_6_] powders, Ketjen black, single-wall carbon nanotubes (SWCNT), and PVDF in a weight ratio of 86:2:2:10 in NMP. The SWCNT and PVDF were pre-mixed in NMP at a mass ratio of 0.4:2:97.6. The resulting mixture was then blade-coated onto an Al sheet (carbon-coated) and dried overnight at 120°C.

**Characterization of the morphology:**

The K discs were sealed in a transparent box, and their surface morphologies were characterized by a three-dimensional confocal optical microscope (Keyence, VHX-7000). ToF-SIMS analysis was conducted using an OrbiSIMS instrument with a primary Bi_3_⁺ beam, a secondary beam voltage of 2 keV, and a secondary beam current of 20 nA. Surface charging was neutralised with the instrument’s electron flood gun. X-ray diffraction (XRD) measurements were performed using a Panalytical X’Pert Pro Thin Film diffractometer. To prevent oxidation during testing, the potassium samples were fully encapsulated with Kapton tape, which does not introduce discernible diffraction artifacts within the measured 2θ range.

**Electrochemical measurements**: Symmetric K//K coin cells (R2032) were assembled in a glove box with an atmosphere of O_2_ and H_2_O below 0.5 ppm. All cells rest for 24 hours before testing. Electrochemical impedance spectroscopy (EIS) was performed on a VSP potentiostat (BioLogic, France) with a frequency range of 10^–2^~10^5^ Hz and a 5 mV AC amplitude. Galvanostatic charge-discharge (GCD) test was performed on a Neware battery cycler (CT-4008).

**Simulations:** Static electric field was simulated using the AC/DC module of the COMSOL Multiphysics software package. A constant voltage difference of 500 mV was applied between the two electrodes, and voltage drop across the medium of the electrodes and electrolyte was computed. Based on experimental observations, the electrodes and electrolyte were specified to the thicknesses of 200 and 670 μm, respectively. To replicate the impact of electrode surface roughness, various curvatures were incorporated into the electrode profile.

**Declaration of Generative AI and AI-assisted technologies in the writing process**

During the preparation of this work the author(s) used ChatGPT Plus to polish the text for clarity and style. After using this tool/service, the author(s) reviewed and edited the content as needed and take(s) full responsibility for the content of the publication.

**Section S1. Morphological evolution of the K surface under repeated rolling.**

Figure S1 summarizes the cumulative impact of repeated roll pressing on the surface morphology and electrochemical behavior of potassium metal. Each rolling pass imposes substantial plastic deformation on the soft K lattice, progressively amplifying surface roughness and generating orange-peel and wrinkle-like features (Fig. S1a–b). As deformation accumulates, localized strain concentrations at grain boundaries promote surface tearing and undulation, consistent with the characteristic plastic flow of alkali metals. Quantitative profilometry reveals that the arithmetic roughness (Ra) increases markedly from 12.4 µm after the first pass to 39.0 µm after the fifth pass (Fig. S1c), underscoring the cumulative nature of deformation-induced degradation.

Electrochemical impedance spectra (Fig. S1d) show a corresponding rise in overall interfacial resistance with increasing pass number, reflecting the enhanced heterogeneity of the electrode/electrolyte interface. Likewise, galvanostatic cycling (Fig. S1e) demonstrates that higher surface roughness leads to intensified voltage noise and reduced stability, as non-uniform current distribution accelerates dendritic growth and interfacial failure. Collectively, these results confirm that repeated rolling inherently deteriorates K surface uniformity and makes the elimination of orange-peel and wrinkle formation extremely difficult through mechanical deformation alone. This provides a clear rationale for adopting the cutting-based fabrication route discussed in the main text.

**Fig. S1.** (a) Schematic illustration showing that multiple rolling passes, required to achieve a target foil thickness, progressively induce surface deformation, evolving from an initial orange-peel texture to severe wrinkling. (b) Optical micrographs of K plates after the 1st, 2nd, 3rd, and 5th rolling passes, showing increased surface roughness and the formation of microcracks and undulations. (c) Evolution of surface roughness (Ra) as a function of rolling pass number. (d) Nyquist plots revealing increased interfacial resistance with successive rolling. (e) Galvanostatic cycling of symmetric cells assembled from K foils rolled with different pass counts, demonstrating increased polarization and reduced stability for more heavily deformed surfaces.

**Section S2. Cutting-based preparation of K@cutting discs.**

Figure S2 illustrates the procedure for preparing K metal discs using the cutting-based fabrication method. A bulk K chunk is first gently scraped to remove the oxidized outer layer and any adventitious contaminants, then trimmed into a rectangular brick with a width of approximately 15 mm. The brick is subsequently mounted in a custom-designed slicing jig that provides mechanical stability and dimensional precision while ensuring safe handling of the highly reactive metal.

A fresh stainless-steel blade is used to slice the brick into thin sheets of uniform and controllable thickness. This subtractive slicing step removes material rather than deforming it, thereby preserving the intrinsic crystallographic orientation and avoiding the strain accumulation associated with roll pressing. Once sliced, 12 mm-diameter discs are punched from the sheets under an inert atmosphere for use in electrochemical cell assembly. This cutting-based route therefore provides smooth, homogeneous K surfaces with minimal mechanical distortion, forming a structurally well-defined baseline for subsequent electrochemical characterization, as discussed in the main text.

**Fig. S2. Cutting-based preparation of K@cutting discs.** A K chunk is first scraped to remove surface contaminants and oxides, then trimmed to a rectangular brick (~15 mm width). The brick is placed in a home-made slicing jig and sliced with a fresh stainless-steel blade to obtain uniform, thin sheets of controlled thickness. Finally, 12 mm-diameter discs are punched from the sheets for cell assembly.

**Section S3. Formation of SEI in electrolytes of different concentrations.**

Figure S3 schematically illustrates the distinct SEI formation pathways in low-concentration (1 M) and high-concentration (4 M) potassium electrolytes. The SEIs on K@rolling and K@cutting electrodes were obtained by immersing the freshly prepared K discs in electrolytes containing potassium bis(fluorosulfonyl)imide (KFSI) dissolved in diethylene glycol dimethyl ether (DEGDME). As depicted in Fig. S3, the SEI composition and structure are jointly determined by the relative contributions of the solvent and the salt anion. In low-concentration electrolytes (1 M KFSI/DEGDME), solvent molecules dominate the primary reduction reactions, producing organic-rich, porous SEIs that are mechanically fragile and prone to continuous breakdown. In contrast, high-concentration electrolytes (4 M KFSI/DEGDME) favor salt-derived decomposition, forming inorganic-rich, ionically conductive SEIs composed mainly of KF, K_2_S, and SO_x_ species.^3, 4^ Such inorganic layers are denser and more chemically stable, effectively suppressing parasitic reactions and dendritic K growth. These contrasting interphase characteristics provide a rational framework for comparing the interfacial stability of K@rolling and K@cutting electrodes discussed in the main text.

**Fig. S3.** **Schematic illustration of SEI formation in low- and high-concentration electrolytes.** The SEI composition depends on both solvent and salt contributions: low-concentration (1 M) electrolytes yield solvent-derived, organic-rich SEIs, whereas high-concentration (4 M) electrolytes promote salt-derived, inorganic-rich SEIs that offer enhanced mechanical robustness and chemical stability.

**Section S4. Electrochemical Impedance and DRT Analyses**

Figure S4 presents the impedance evolution and DRT deconvolution corresponding to the cells discussed in Fig. 2. Before cycling (Fig. S4e), all electrodes show comparable interfacial profiles, but the 4 M systems exhibit significantly lower total resistance (R_SEI_ ≈ 40–60 Ω, R_ct_ ≈ 1300–1600 Ω) compared with 1 M cells (R_SEI_ ≈ 200 Ω, R_ct_ ≈ 8000 Ω), reflecting a more compact initial SEI. Upon cycling (Fig. S4a–d), impedance growth is most pronounced for K@rolling-1M, consistent with rapid SEI degradation and irregular ion transport. K@cutting-1M shows a slower increase, indicating that the smoother surface mitigates but does not eliminate surface film instability. Conversely, both 4 M systems maintain much lower and more stable resistance, with K@cutting-4M exhibiting nearly constant values (R_SEI_ ≈ 38 Ω, R_ct_ ≈ 350 Ω) through nine cycles, as confirmed by DRT fits (Fig. S4f–g).

**Fig. S4. Electrochemical impedance and DRT analyses of symmetric K cells.** a–d) Nyquist plots of K@rolling-1M, K@cutting-1M, K@rolling-4M, and K@cutting-4M from the pre-cycle to the 9th cycle, showing the progressive impedance evolution. (e) Comparative Nyquist plots at the pre-cycle stage, highlighting the significantly lower total resistance in 4 M electrolytes. (f–g) Fitted DRT spectra separating surface-film resistance (R_SEI_) and charge-transfer resistance (R_ct_). (h) Comparative Nyquist plots at the 9th cycle, illustrating the suppression of impedance growth for K@cutting-4M due to its smooth surface and robust SEI.

**Fig. S5. Enlarged DRT spectra of (a) K@rolling-4M and (b) K@cutting-4M from the 1^st^ to 9^th^ cycle.**

**Section S5. Determination of Exchange Current Density (j₀) from Tafel Analysis**

To evaluate the interfacial charge-transfer kinetics of potassium plating and stripping, Tafel analyses were conducted on symmetric K||K cells assembled with K@rolling-1M, K@cutting-1M, K@rolling-4M, and K@cutting-4M electrodes. The overpotential (η) and current density (j) obey the Tafel relationship:

$$\eta=a+b\log j$$

where *a* and *b* are the intercept and Tafel slope, respectively. The exchange current density **(**j₀**)** is extracted by extrapolating the linear fit of the η–log j curve to η = 0, using:

$$j_{0}={10}^{-\frac{a}{b}}$$

Fittings were performed within the low-overpotential region (50–150 mV), where the logarithmic dependence holds.

The derived kinetic parameters are summarized in **Table S1**.

The exchange current density (j₀) systematically increases from K@rolling-1M to K@cutting-4M, indicating a continuous improvement in interfacial charge-transfer kinetics. The surface refinement achieved by the cutting process reduces geometric irregularities and ensures uniform electric-field distribution, while the 4 M electrolyte forms a robust, inorganic rich SEI that minimizes interfacial polarization. The highest j₀ value (4.1 × 10⁻^3^ mA cm⁻^2^) of K@cutting-4M demonstrates that the combination of crystallographic homogeneity and SEI robustness enables the most efficient K⁺ exchange at the metal–electrolyte interface, consistent with the reduced overpotential observed in Fig. 2m and the stable cycling performance shown in Fig. 2n–p.

**Table S1**. Tafel parameters and calculated exchange current densities (j₀) for K metal electrodes

| Electrode | Tafel slope  (*b*, V dec⁻¹) | Intercept  (*a*, V) | log j₀ | j₀  (mA cm⁻^2^) |
| --- | --- | --- | --- | --- |
| K@rolling-1M | 0.153 | −3.61 | −2.60 | 2.5 × 10⁻^3^ |
| K@cutting-1M | 0.137 | −3.55 | −2.59 | 2.6 × 10⁻^3^ |
| K@rolling-4M | 0.121 | −3.50 | −2.50 | 3.2 × 10⁻^3^ |
| K@cutting-4M | 0.108 | −3.43 | −2.39 | 4.1 × 10⁻^3^ |

**Fig. S6. Determination of Exchange Current Density (j₀) from Tafel Analysis**. Linear regions of Tafel plots were fitted to extract j₀ values for K@rolling-1M, K@cutting-1M, K@rolling-4M, and K@cutting-4M, showing progressively improved charge-transfer kinetics with surface smoothing and SEI strengthening.

**Section S6. In situ optical microscopy characterization.**

A home-made optical microscopy cell was designed to enable real-time visualization of K plating morphology. The cell consists of a rectangular PEEK housing with a central observation window, sealed by two glass windows and compressed using stainless-steel tightening rods. Symmetric K||K cells assembled in this device were operated at 0.5 mA cm⁻^2^ to monitor the evolution of morphology during continuous plating.

Extending the plating duration to 30–60 minutes further amplified the morphology trends observed at shorter times. The K@rolling-1M and K@rolling-4M electrodes exhibited significant thickening of the deposited layer, with increasingly pronounced dendritic protrusions and side-branching instabilities. These effects were particularly severe in the 1 M electrolyte, where the low-strength SEI and deformation-induced surface asperities jointly promoted localized current concentration. By contrast, both K@cutting-1M and K@cutting-4M electrodes maintained markedly more stable and uniform morphologies. Although K@cutting-1M produced a relatively loose and porous deposit, its growth remained spatially homogeneous with minimal dendritic projection. The K@cutting-4M electrode displayed the most compact and dense deposition among all samples, with a continuous and conformal layer that remained stable throughout the extended plating period.

**Fig. S7.** Extended-time in situ optical microscopy of K plating using a home-made optical cell. (a–d) Optical microscopy images showing the deposition morphology after 60 minutes for (a) K@rolling-1M, (b) K@cutting-1M, (c) K@rolling-4M, and (d) K@cutting-4M.

**Section S7. Spatial Distribution of SEI Components from 2D ToF-SIMS Maps**

To deconvolute the spatial heterogeneity of SEI formation, the SEI was segmented into four depth-defined regions based on the evolution of secondary-ion intensities during sputtering: the outer layer is defined by a higher abundance of organic species (C_2_H⁻) compared to inorganic species such as KF_2_⁻, KSO_3_⁻, S⁻, and KCO₃⁻ at the surface. As the etching depth increases, the proportion of inorganic species gradually rises and eventually exceeds that of the organic components. The middle layer is characterized by a gradual decrease in the proportions of inorganic species (KF_2_⁻, KSO_3_⁻, S⁻, and KCO_3_⁻), while K(OH)_2_⁻ reaches its peak concentration and K_3_⁻ increases to approximately 25%. The inner layer is defined by a K_3_⁻ proportion ranging from 25% to 75%. Finally, the bulk layer corresponds to a region where the proportion of K_3_⁻ exceeds 75%.

To better visualize the spatial distribution of individual species, 2D overlap maps of C_2_H⁻, KF_2_⁻, and K(OH)_2_⁻ at specific etching depths corresponding to the outer, middle, and inner SEI layers are presented in **Fig. S8**. Before cycling, the outer layer (1–13s) of K@cutting-1M shows a uniform distribution of C_2_H⁻, whereas K@cutting-4M exhibits uniformly dispersed KF_2_⁻ clusters embedded within the C_2_H⁻ matrix. In the middle layer of both samples, KF_2_⁻ dominates with a relatively uniform distribution. The inner layer displays a pronounced K(OH)_2_⁻ signal, attributed to the pristine native layer on the K surface. After cycling, K@cutting-1M exhibits significant signal attenuation and a more disordered distribution of SEI components, indicative of repeated SEI fracture and reformation, which contributes to non-uniform potassium plating morphology. In contrast, K@cutting-4M retains a well-defined, layered SEI structure, characterized by a consistent C_2_H⁻ signal at the surface and stable, uniformly distributed inorganic species in the deeper layers. This structural integrity highlights the enhanced SEI durability afforded by the higher electrolyte concentration, which effectively promotes uniform K plating/stripping and suppresses dendrite formation during repeated cycling.

**Fig. S8.** 2D overlap maps of C_2_H^−^, KF_2_^−^, and K(OH)_2_^−^ at sputtering depths corresponding to the outer, middle, and inner SEI regions for K@cutting-1M and K@cutting-4M.

**Figure S9. Structural and morphological of K_1.97_Mn[Fe(CN)_6_].** (a) XRD pattern confirming the crystalline phase of the as-synthesized K_1.97_Mn[Fe(CN)_6_], with diffraction peaks matching well with the characteristic reflections of Prussian blue analogues, indicating high phase purity and a well-defined cubic framework. (b) SEM image showing the representative morphology and particle size distribution. The sample consists of uniformly distributed submicron particles with a cubic-like morphology, forming aggregated clusters, which is typical for Prussian blue analogue materials synthesized via co-precipitation

**Fig. S10.** The voltage profiles at different cycles of K@cutting||K_1.97_Mn[Fe(CN)_6_] full cell in 4M electrolyte.

**Fig. S11.** Long-term cycling stability of the contrast K@rolling||K_1.97_Mn[Fe(CN)_6_] full cell in 4M electrolyte at a current density of 0.5 mA cm^−2^ and high capacity of 0.85 mA h cm^−2^. During cycling, occasional micro-short circuits at the K anode lead to abnormal voltage behavior, preventing the cell from reaching the preset upper cutoff voltage of 4.5 V during charging. To ensure consistent testing and avoid overcharging under these unstable conditions, a capacity cutoff limit (150 mAh g⁻^1^) was applied during charging in those cycles.

**Table S2**. The comparison of K||K cycle life between K@cutting-4M and reported K metal anodes

| Anodes | Current/capacity  (mA cm^−2^/ mA h cm^−2^) | Cycling life  (hour) | Ref. |
| --- | --- | --- | --- |
| KxPy@K | 0.5-0.5 | 550 | ^5^ |
| [K@DN-MXene-CNT](mailto:K@DN-MXene-CNT) | 0.5-0.5 | 300 | ^6^ |
| CC@SnO2 | 0.2-1 | 635 | ^7^ |
| Co−CNF@K | 0.5-0.5 | 1300 | ^8^ |
| PPS-treated K | 1-1 | 1100 | ^9^ |
| K-GIC | 0.4-0.4 | 700 | ^10^ |
| [K@PTFE](mailto:K@PTFE) | 0.5-0.5 | 1000 | ^11^ |
| CBC-K | 1-1 | 1100 | ^12^ |
| K@K2Te | 0.5-0.5 | 800 | ^13^ |
| SA-Co@HC-K | 0.5-0.5 | 2500 | ^14^ |
| [K@CNTs](mailto:K@CNTs) | 1-1 | 222 | ^15^ |
| MSCNF-K | 1-1 | 233 | ^16^ |
| K@NGM | 1-1 | 220 | ^17^ |
| CoZn@HCT | 0.5-0.5 | 1200 | ^18^ |
| K-Co-NOC-CNM | 1-1 | 1000 | ^19^ |
| [K@MES](mailto:K@MES) | 0.5-0.5 | 2400 | ^20^ |
| [TS-PKS@K](mailto:TS-PKS@K) | 0.5-0.5 | 2900 | ^21^ |
| KF-Zn@K | 0.5-0.5 | 2100 | ^22^ |
| [K@PMCFs](mailto:K@PMCFs) | 0.5-0.5 | 3200 | ^23^ |
| [ZnTe@PCNF-K](mailto:ZnTe@PCNF-K) | 0.5-0.5 | 3100 | ^24^ |
| [**K@cutting-4M**](mailto:K@mesh) | **0.5-4** | **4000** | **This work** |

| **Methods** | **Atomic (%)** | | |
| --- | --- | --- | --- |
| MP-AES | K | Mn | Fe |
|  | 1.97 | 1 | 1 |
| Elemental Analysis | C | N | H |
|  | 1.69 | 1.68 | 0 |

**Table S3.** Elemental analysis and MP-AES data of as synthesized K_1.97_Mn[Fe(CN)_6_] powder

**References**

1.	Deng, L.; Qu, J.; Niu, X.; Liu, J.; Zhang, J.; Hong, Y.; Feng, M.; Wang, J.; Hu, M.; Zeng, L., Defect-free potassium manganese hexacyanoferrate cathode material for high-performance potassium-ion batteries. *Nat. Commun.* **2021,** *12* (1), 2167.

2.	Hosaka, T.; Kubota, K.; Kojima, H.; Komaba, S., Highly concentrated electrolyte solutions for 4 V class potassium-ion batteries. *Chem. Commun. (Cambridge, U. K.)* **2018,** *54* (60), 8387-8390.

3.	Xiao, N.; McCulloch, W. D.; Wu, Y., Reversible Dendrite-Free Potassium Plating and Stripping Electrochemistry for Potassium Secondary Batteries. *J. Am. Chem. Soc.* **2017,** *139* (28), 9475-9478.

4.	Gao, Y.; Hou, Z.; Zhou, R.; Wang, D.; Guo, X.; Zhu, Y.; Zhang, B., Critical roles of mechanical properties of solid electrolyte interphase for potassium metal anodes. *Adv. Funct. Mater.* **2022,** *32* (17), 2112399.

5.	Shi, P.; Zhang, S.; Lu, G.; Wang, L.; Jiang, Y.; Liu, F.; Yao, Y.; Yang, H.; Ma, M.; Ye, S., Red phosphorous‐derived protective layers with high ionic conductivity and mechanical strength on dendrite‐free sodium and potassium metal anodes. *Adv. Energy Mater.* **2021,** *11* (5), 2003381.

6.	Tang, X.; Zhou, D.; Li, P.; Guo, X.; Sun, B.; Liu, H.; Yan, K.; Gogotsi, Y.; Wang, G., MXene‐based dendrite‐free potassium metal batteries. *Adv. Mater.* **2020,** *32* (4), 1906739.

7.	Qiao, F.; Meng, J.; Wang, J.; Wu, P.; Xu, D.; An, Q.; Wang, X.; Mai, L., Building carbon cloth-based dendrite-free potassium metal anodes for potassium metal pouch cells. *Journal of Materials Chemistry A* **2021,** *9* (40), 23046-23054.

8.	Wang, L.; Wang, H.; Cheng, M.; Hong, Y.; Li, M.; Su, H.; Sun, J.; Wang, J.; Xu, Y., Metal–organic framework@ polyacrylonitrile-derived potassiophilic nanoporous carbon nanofiber paper enables stable potassium metal anodes. *ACS Applied Energy Materials* **2021,** *4* (6), 6245-6252.

9.	Park, J.; Jeong, Y.; Alfaruqi, M. H.; Liu, Y.; Xu, X.; Xiong, S.; Jung, M.-G.; Jung, H.-G.; Kim, J.; Hwang, J.-Y., Stable solid electrolyte interphase for long-life potassium metal batteries. *ACS Energy Letters* **2021,** *7* (1), 401-409.

10.	Zhang, J.; Li, Y.; Zhu, L.; Wang, X.; Tu, J., An intercalation compound for high-safe K metal batteries. *Energy Storage Materials* **2021,** *41*, 606-613.

11.	Qin, C.; Wang, D.; Liu, Y.; Yang, P.; Xie, T.; Huang, L.; Zou, H.; Li, G.; Wu, Y., Tribo-electrochemistry induced artificial solid electrolyte interface by self-catalysis. *Nat. Commun.* **2021,** *12* (1), 7184.

12.	Zhou, M.; Qi, W.; Hu, Z.; Cheng, M.; Zhao, X.; Xiong, P.; Su, H.; Li, M.; Hu, J.; Xu, Y., Highly potassiophilic carbon nanofiber paper derived from bacterial cellulose enables ultra-stable dendrite-free potassium metal anodes. *ACS Appl. Mater. Interfaces* **2021,** *13* (15), 17629-17638.

13.	Yang, H.; He, F.; Li, M.; Huang, F.; Chen, Z.; Shi, P.; Liu, F.; Jiang, Y.; He, L.; Gu, M., Design principles of sodium/potassium protection layer for high‐power high‐energy sodium/potassium‐metal batteries in carbonate electrolytes: a case study of Na2Te/K2Te. *Adv. Mater.* **2021,** *33* (48), 2106353.

14.	Zhang, D.; Ma, X.; Wu, L.; Wen, J.; Li, F.; Zhou, J.; Rao, A. M.; Lu, B., Coupling Low‐Tortuosity Carbon Matrix with Single‐Atom Chemistry Enables Dendrite‐Free Potassium‐Metal Anode. *Adv. Energy Mater.* **2023,** *13* (2), 2203277.

15.	Yang, Y.; Huang, C.; Zhang, Y.; Wu, Y.; Zhao, X.; Qian, Y.; Chang, G.; Tang, Q.; Hu, A.; Chen, X., Processable Potassium–Carbon Nanotube Film with a Three-Dimensional Structure for Ultrastable Metallic Potassium Anodes. *ACS Appl. Mater. Interfaces* **2022,** *14* (50), 55577-55586.

16.	Li, S.; Zhu, H.; Liu, Y.; Han, Z.; Peng, L.; Li, S.; Yu, C.; Cheng, S.; Xie, J., Codoped porous carbon nanofibres as a potassium metal host for nonaqueous K-ion batteries. *Nat. Commun.* **2022,** *13* (1), 4911.

17.	Xiong, J.; Ye, M.; Wang, Z.; Chen, J.; Zhang, Y.; Tang, Y.; Li, C. C., Fast and homogeneous ion regulation toward a 4 V, high-rate and dendrite-free potassium metal battery. *Chem. Eng. J.* **2022,** *442*, 135927.

18.	Cheng, G.; Liu, S.; Wang, X.; Li, X.; Su, Y.; Shi, J.; Huang, M.; Shi, Z.; Wang, H.; Yan, Z., CoZn nanoparticles@ hollow carbon tubes enabled high-performance potassium metal batteries. *ACS Appl. Mater. Interfaces* **2022,** *14* (40), 45364-45372.

19.	Zhao, L. K.; Gao, X. W.; Mu, J.; Luo, W. B.; Liu, Z.; Sun, Z.; Gu, Q. F.; Li, F., Durable integrated K‐metal anode with enhanced mass transport through potassiphilic porous interconnected mediator. *Adv. Funct. Mater.* **2023,** *33* (41), 2304292.

20.	Ding, H.; Wang, J.; Zhou, J.; Wang, C.; Lu, B., Building electrode skins for ultra-stable potassium metal batteries. *Nat. Commun.* **2023,** *14* (1), 2305.

21.	Zhang, D.; Liu, M.; Shi, W.; Qiu, Y.; Hu, Y.; Yuan, Z.; Xue, H.; Kong, L.; Zhao, K.; Ren, J., Dual‐Gradient Engineering of Conductive and Hierarchically Potassiophilic Network for Highly Stable Potassium Metal Anode. *Adv. Energy Mater.* **2024,** *14* (40), 2401960.

22.	Zhao, L.-K.; Gao, X.-W.; Gu, Q.; Ge, X.; Ding, Z.; Liu, Z.; Luo, W.-B., Realizing a dendrite-free metallic-potassium anode using reactive prewetting chemistry. *eScience* **2024,** *4* (2), 100201.

23.	Chen, Z.; Wang, L.; Zheng, J.; Huang, Y.; Huang, H.; Li, C.; Shao, Y.; Wu, X.; Rui, X.; Tao, X., Unraveling the Nucleation and Growth Mechanism of Potassium Metal on 3D Skeletons for Dendrite-Free Potassium Metal Batteries. *ACS Nano* **2024,** *18* (11), 8496-8510.

24.	Chen, X.; Zhang, M.; Jia, L.; Yi, Y.; Su, Y.; Lian, X.; Lu, M.; Zou, Y.; Chen, Z.; Wang, Y., Alloy‐Boosted Potassiophilic Membrane Interphase for Ultrastable K Metal Anodes. *Adv. Funct. Mater.* **2024**, 2421110.
